# Economic analysis of cloud-based desktop virtualization implementation at a hospital

**DOI:** 10.1186/1472-6947-12-119

**Published:** 2012-10-30

**Authors:** Sooyoung Yoo, Seok Kim, TaeKi Kim, Rong-Min Baek, Chang Suk Suh, Chin Youb Chung, Hee Hwang

**Affiliations:** 1 Center for Medical Informatics, Seoul National University Bundang Hospital, 166, Gumi-ro, Bundang-gu, Seongnam-si, South korea; 2 Department of Plastic Surgery, Seoul National University Bundang Hospital, 166, Gumi-ro, Bundang-gu, Seongnam-si, South korea; 3 Department of Obstetrics and Gynecology, Seoul National University Bundang Hospital, 166, Gumi-ro, Bundang-gu, Seongnam-si, South korea; 4 Department of Orthopedics, Seoul National University Bundang Hospital, 166, Gumi-ro, Bundang-gu, Seongnam-si, South korea; 5 Department of Pediatrics, Seoul National University Bundang Hospital, 166, Gumi-ro, Bundang-gu, Seongnam-si, South korea

**Keywords:** Desktop virtualization, Economic analysis, Hospital information system

## Abstract

**Background:**

Cloud-based desktop virtualization infrastructure (VDI) is known as providing simplified management of application and desktop, efficient management of physical resources, and rapid service deployment, as well as connection to the computer environment at anytime, anywhere with anydevice. However, the economic validity of investing in the adoption of the system at a hospital has not been established.

**Methods:**

This study computed the actual investment cost of the hospital-wide VDI implementation at the 910-bed Seoul National University Bundang Hospital in Korea and the resulting effects (i.e., reductions in PC errors and difficulties, application and operating system update time, and account management time). Return on investment (ROI), net present value (NPV), and internal rate of return (IRR) indexes used for corporate investment decision-making were used for the economic analysis of VDI implementation.

**Results:**

The results of five-year cost-benefit analysis given for 400 Virtual Machines (VMs; i.e., 1,100 users in the case of SNUBH) showed that the break-even point was reached in the fourth year of the investment. At that point, the ROI was 122.6%, the NPV was approximately US$192,000, and the IRR showed an investment validity of 10.8%. From our sensitivity analysis to changing the number of VMs (in terms of number of users), the greater the number of adopted VMs was the more investable the system was.

**Conclusions:**

This study confirms that the emerging VDI can have an economic impact on hospital information system (HIS) operation and utilization in a tertiary hospital setting.

## Background

Virtualization is an abstract concept that requires the system’s hardware, the physical operation system, to exist and operate in a virtual space [1-3]. Virtual Desktop Infrastructure (VDI) system based on virtualization and cloud computing technologies allows users to connect to their computing environment and use the information system from wherever the internet is available without being limited by connecting devices or by time and place. However, despite the interest in this new technology due to its advantages in terms of efficient management of physical resources and rapid service deployment [4-6], actual implementations are not yet common. This may be because of the uncertain outcomes of the invested capital, despite the theoretical effects of the new technology. According to a multiple-response survey of U.S. hospital managers, unclear return on investment (ROI) was reported as one of the major reasons for not investing in the information system [7].

Although there have been analyses of the investment in VDI and its effects on a single hospital information system (HIS), these reports have mainly focused only on outcome effects with no comparison with investment cost [8,9]. No study has analyzed the validity of investing in VDI from an economic perspective that considered the actual investment cost. Thus, this study intended to economically analyze the effects arising from adopting cloud-based VDI at a 910-bed, general university hospital through a comparison with the actual investment cost.

## Methods

### Study subject

The study subject was Seoul National University Bundang Hospital (SNUBH) located in Seoul metropolitan area (Seongnam City, Gyeonggi-do). SNUBH, a public tertiary hospital that has been completely digital since May 2003, was equipped with a comprehensive electronic health record (EHR) system with Stage 7 from the Healthcare Information and Management Systems Society (HIMSS) Analytics Electronic Medical Record Adoption Model (EMRAM) in October 2010 [10]. The hospital has 910 beds. Approximately 542 doctors, 919 nurses, and 277 staff members were employed.

### VDI implementation method

SNUBH launched a VDI solution in November 2011 after implementing it over a period of approximately 4 months, beginning in July 2011. The implementation allowed all hospital information systems, including Electronic Medical Record (EMR), Picture Archiving and Communications System (PACS), and other clinical and administrative applications, to be accessed from all types of devices (including desktops, iOS-based table PCs, Androd-based table PCs, and laptops). It can be accessible by 1,100 people through 400 Virtual Machines (VMs). Issues related to system architecture and security and privacy aspects have been solved through the dual implementation of server architecture and Active Directory (AD) based authorization. Virtual Private Network (VPN) was used for secure outside access. Table 1 shows a summary list of hardware and software components that comprised the VDI.

**Table 1:** List of hardware and software that composed the VDI

**Item**	**Specification**	**Number**
**Hardware**		
Mgt. Access Server	CPU : 2.93GHz, 12Core / Memory : 96GB	3
VDI Server	Same as above	18
Network	Storage Area Network (SAN), L4, L2	Dual
VPN	Concurrent 200 user	Dual
Storage	FC 60TB	1
Tablet PC	iPad 16GB	400
**Software**		
VDI Solution	Concurrent 400user / VMware / Oracle DBMS	1
License of Virtual OS	Virtual OS Microsoft Virtual Desktop Access	1
Mobile Device	Mobile only App.	1

### Analysis method

#### Assumptions

Table 2 summarizes the assumptions this study adopted for the economic analysis. Information system was assumed to last five years due to the rapid advancement of information technology [4,11,12]. According to VMware’s reports that VDI use reduces the time spent on account management from 15 to 5 minutes and reduces the number of help desk calls by 92% [8,9], this study assumed that the rate of decrease of time spent and the rate of service demand decrease were be 20% and 10%, respectively.

**Table 2:** Assumptions for economic analysis

**Classification**	**Assumptions**
Operation period	2012 to 2016 (five years)
Annual effects	Effects occur in the same manner every year. The expected effect occurring after the operation period is not reflected.
Work utilization	All users at SNUBH were assumed to utilize VDI beginning in 2012.
Discount rate	4.66%^a^
Exchange rate	US$1= KRW 1,117.5^b^
ROI	Calculated based on the present value.
Present value computation	Discounted cash flow (DCF) was used.
Rate of decrease of time spent	20% decrease from the previous year
	-Average recovery time due to reduced PC errors and difficulties
	-Average update time due to reduced application and OS update time
	-Average time taken resulting from reduced account management time
Rate of service demand decrease	10% decrease from the previous year
	- Service demand due to reduced PC errors and difficulties

^a^The discount rate was set as 4.66%, which was the average interest rate of corporate bonds that Bank of Korea reported in 2010.

^b^It was an exchange rate that announced by the Korea Exchange Bank on March 8, 2012.

#### Cost items determination

Considering the cost items of previous studies on VDI [13], EMR [11], and Computerized Physician Order Entry (CPOE) [14] implementations, this study classified costs items into initial implementation costs and the operation and maintenance costs. The initial implementation costs consisted of hardware costs, software costs, and initial outsourcing implementation costs, and the operation and maintenance costs consisted of ongoing support and maintenance costs and software annual license costs (for the use of virtual desktop access). See Additional file 1 for the investment costs in detail.

#### Benefit items determination

Lipsitz et al. [13] identified outsourcing savings, help desk savings, U.S. contractor savings, avoided additional IT hires, avoided PC purchases, and office space savings as the six benefits of VDI in conjunction with Thin Client implementation. According to VMware [9], a hospital that implemented VDI reported reduced operation costs and reduced help desk calls as the benefit items. See Additional file 1 for the estimated benefit revenue.

Of the benefit items used in previous studies, this study used only those items that were applicable to cases in which only VDI was implemented without Thin Client. The benefit items selected for this study are as follows.

##### Benefits Due to reduced PC errors and difficulties (consulting rooms)

The errors and difficulties associated with the computers used in consulting rooms are an impediment to outpatient treatment in an environment in which all treatment data are digitalized, and this is directly related to the decrease in the hospital’s consultation fee income. Hence, the effects of reduced PC errors and difficulties and shortened recovery time resulting from VDI implementation are benefits resulting from the increased outpatient turnover ratio. The formula used to compute the benefits is as follows:

*Formula: {(Service demand due to PC errors and difficulties in consulting rooms in 2010 x Average recovery time per occurrence) – (Service demand reduced by 10% x Average recovery time per occurrence reduced by 20%)} x Average outpatient consultation fee per minute in 2010 x 0.75* (The formula is adjusted by multiplying by 0.75 because consulting rooms were open for at least 6 hours of an 8-hour day in this study).

##### Benefits Due to reduced PC errors and difficulties (places other than consulting rooms)

The effects of reduced PC errors and difficulties in places other than consulting rooms was computed as the opportunity cost arising from the reduction in the administrator’s administrative time, a cost-savings effect.

Formula: {(Service demand due to errors and difficulties in places other than consulting rooms in 2010 x Average recovery time per occurrence) – (Service demand reduced by 10% x Average recovery time per occurrence reduced by 20%)} x Average wage per minute for the administrator in 2010.

##### Benefits Due to reduced update time for applications and OS

Many types of application and operating systems used require periodical or constant updates, which are directly related to the system administrative cost. In this study, the number of updates does not change due to VDI implementation, but the update administration time is expected to be reduced, and the opportunity cost of the decrease in administrator time used for updates is computed. This is a cost-savings effect.

Formula: {(Number of updates in 2010 x Average time for one update) – (Number of updates in 2010 x Average time for one update reduced by 20%)} x Average wage per minute for the administrator in 2010.

##### Benefits Due to reduced account management time

Hiring new hospital employees generates the task of initializing the computer to be used for work. In VDI, computer initialization is simplified through a simple VDI account addition. This study computed this as the opportunity cost for the decrease in the administrator’s time. This is a cost-savings effect.

Formula: {(Number of account management occurrences in 2010 x Average time per occurrence) – (Number of account management occurrences in 2010 x Average time per occurrence reduced by 20%)} x Average administrator wage per minute in 2010.

#### Analysis measures

The selected cost items and benefit items were analyzed using ROI, net present value (NPV), and the internal rate of return (IRR), which are indexes that are commonly used to calculate the effects of corporate investments [15].

#### Sensitivity analysis

A sensitivity analysis was performed to examine the effects that a variety of future fluctuations could have on the benefits of VDI implementation [15].

##### Sensitivity analysis based on changes in the total amount of costs and benefits and the discount rate

Changes in the ROI, NPV, and IRR were analyzed according to the change in the total cost and benefit amount over five years and in the discount rate in effect. Changes resulting from a ±20% change in each item were analyzed.

##### Sensitivity analysis based on the number of VMs

Changes in the ROI, NPV, and IRR based on the number of adopted VMs were analyzed. The investment amount and the value of the ensuing benefits changed with the number of VMs (in terms of number of users). In case of the initial implementation cost, the hardware cost for 60TB of storage was adjusted, and the software cost for 40 VM was adjusted. For operation and maintenance costs, the software annual license (for the use of virtual desktop access) for 40 VMs was adjusted for each year, and the cost of ongoing support and maintenance for the adjusted hardware and software was adjusted for the sensitivity analysis.

## Results

### Customization issues during VDI implementation

VDI implementation requires customization to each hospital’s particular environment. The following issues manifested in SNUBH’s customization process: 1) determining the appropriate number of VMs to be adopted for the given number of VDI users and distinguishing between personal users and public users for resource distribution; 2) determining where to pursue dualization for stability of use; 3) the implementation scale of the ensuring hardware.

### Results of the five-year cost-benefit analysis

**Figure 1:**
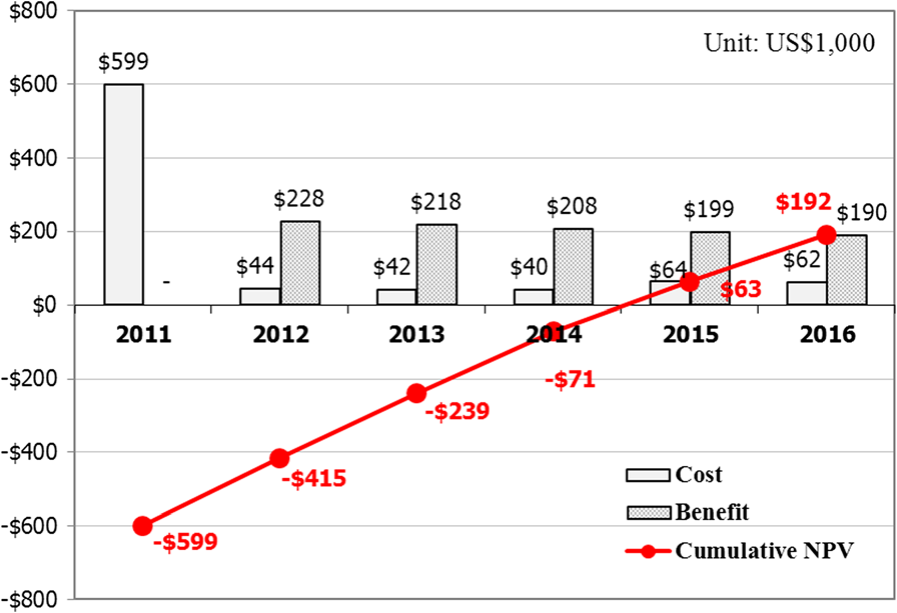
Five-year economic analysis of VDI.

Figure 1 shows the results of cost-benefit analysis when VDI is assumed to be operated for five years. The present value was computed using 4.66%, which is the Bank of Korea’s 2010 average interest rate for corporate bonds, as the discount rate for the costs and benefits of each year, except for the first year, for which the DCF was used.

As can be seen in Figure 1, the implementation cost occurred in 2011 and the operation and maintenance costs occur each year thereafter. Benefits occurred every year since 2012, when VDI was launched. In 2015, the fourth year after the VDI launch, the accumulative NPV is greater than 0, marking the transition from loss to gain. According to the results of the five-year cost-benefit analysis, the ROI is 122.6%, higher than 100%, which indicates a highly effective investment, and the IRR is 10.8%, higher than the discount rate of 4.66%, which also indicates an effective investment. This can mean that approximately a $192,000-effect will be generated over five years based on the current dollar value.

### Sensitivity analysis results

#### Results of the sensitivity analysis based on the changes in the total costs and benefits amount and the discount rate

The effects of ±20% changes in the total costs and benefits amounts and the discount over the five-year period on the ROI, NPV, and IRR were examined, and the results are shown in Table 3. As shown in Table 3, when the benefits were reduced by 20% and 10% and when the costs were increased by 20%, the investment was not validated. In particular, there was a loss when the benefits were reduced by 20%; the ROI was less than 100% (82%), the NPV showed a deficit of approximately US$147,000, and the IRR was less than 4.66% (−10.0%) over the five-year period. Given future fluctuations, ROI, NPV, and IRR were the highest when the benefits were increased by 20%; over the five-year period, the ROI was 170.1%, the NPV was approximately US$597,000, and the IRR was 29.3%.

**Table 3:** Sensitivity analysis based on the total amount fluctuations. (Unit: US$1,000)

**Classification**		**ROI**	**NPV**	**IRR**
Costs	80%	153.2%	363	23.9%
	90%	136.2%	277	16.8%
	100%	122.6%	192	10.8%
	110%	111.4%	107	5.6%
	120%	102.1%	22	1.1%
Benefits	80%	82.7%	−147	−10.0%
	90%	101.7%	14	0.9%
	100%	122.6%	192	10.8%
	110%	145.4%	386	20.2%
	120%	170.1%	597	29.3%
Discount rate(4.66%)	80%	124.7%	212	11.8%
	90%	123.6%	202	11.3%
	100%	122.6%	192	10.8%
	110%	121.5%	182	10.3%
	120%	120.5%	173	9.8%

#### Results of sensitivity analysis based on the number of VMs

The number of VMs changes according to the size of the hospital implementing VDI and the number of employees (users). This change is examined through sensitivity analysis.

Table 4 shows changes in the ROI, NPV, and IRR when the number of VMs is adjusted in increments of 40 from 400. The larger the number of adopted VMs was, the greater the ROI, NPV, and IRR values were. In addition, when the number of VMs was greater than 320, the ROI was always above 100%, the NPV was always greater than 0, and the IRR was higher than the 4.66% discount rate throughout the five-year period, which implies that the investment was valid. In cases in which the adopted VMs numbers were 240 and 280, the ROI and NPV values did not indicate a deficit, but the IRR was lower than the discount rate of 4.66% in the five-year period, which means that the investment was not valid. Furthermore, when the number of VMs was 200, all indexes indicated a deficit.

**Table 4:** Sensitivity analysis based on the number of vms. (Unit: US$1,000)

**Number of VMs**	**ROI**	**NPV**	**IRR**
200	92.0%	−45	−3.8%
240	100.4%	2	0.2%
280	107.3%	50	3.5%
320	113.2%	97	6.3%
360	118.2%	145	8.7%
400	122.6%	192	10.8%
440	126.4%	240	12.7%
480	129.7%	287	14.3%
520	132.7%	335	15.8%
560	135.4%	382	17.1%
600	137.8%	430	18.3%

## Discussion

This study economically analyzed the validity of investing in the implementation of VDI at a hospital on the basis of actual investment costs of SNUBH. Previous economic analyses of VDI implementation at a hospital did not consider investment costs; they reported only the outcomes of the VDI implementation. For example, Huntsville Hospital in the United States reported such effects as an electricity savings of 72%, decreased hardware costs and operating costs, improved speed, reduced user account management time (from 15 to 5 minutes), and enhanced security when VDI was adopted together with Thin Client [8]. Norton Healthcare of the United States reported that the number of help desk calls decreased by 92% with VDI implementation, and resource management time was also reduced because resources were centrally managed with no user interference [9].

Regarding economic analysis of VDI implementation in conjunction with Thin Client at an ordinary company, Lipsitz et al. [13] argued that if VDI were operated for four years at a company, an ROI of up to 290% would be achieved; considering the future risks, the ROI, NPV, and IRR would be 255%, US$7,968,140, and 178%, respectively, reaching the break-even point after 17 months and yielding a profit thereafter. They also reported that the costs would be US$3,124,263.

The results of this study on the economic analysis of VDI at a hospital indicated that if 400 VMs and VDI were operated for five years at a 910-bed tertiary hospital, the investment would be effective, with an ROI of 122.6%, an NPV of approximately US$192,000, and an IRR of 10.8%. The amount invested over the five-year period is approximately US$852,000, and approximately US$1,044,000 in benefits is generated. The effects are greater as the number of implemented VMs (in terms of number of users) grows; when the number of VMs is less than 320, the ROI is low or reflects a loss.

Now we would like to note that the cost savings and revenue gains resulted from the economic analysis using the actual investment cost were estimated and not actual. The further study will be continued to evaluate actual ROI using actual benefit data after operating VDI for five years in our hospital in the future.

This study is different in that it was done in a general university hospital setting and was restricted to cases of VDI-only implementation in no conjunction with Thin Client that can further reinforce the effects of VDI if VDI is implemented in Thin Client environment. Although this study has a limitation in that it was conducted in a single hospital and did not consider cultural differences in healthcare environment, we believe that the findings of this study would be meaningful and valuable in consideration of utilization of the emerging VDI technology in a healthcare environment.

## Conclusions

Recent advances in information technology have led to vast improvements in HIS, and new systems are constantly under development with the goal of providing more accurate and safer medical services. Although the new systems’ efficacies are accepted, there are many difficulties involved in making the decision to invest in a new system [7]. This study economically analyzed the investment effects of VDI implementation at SNUBH, a hospital that is gaining attention for its use of emerging technologies. The results of our study confirmed that VDI can have an economic impact on HIS operation and utilization, with ROI of 122.6% for the option of 400 VMs and five-year’s operation period.

This study involved only a quantitative analysis of the economic aspects of VDI, but qualitative effects could also be a crucial factor in investment decision making. A study on real management with regard to qualitative effects may be necessary in the future.

## Competing interests

There are no conflicts of interests that could inappropriately influence the authors’ findings.

## Authors’ contributions

S. Yoo and S. Kim designed the study, analyzed the data, and drafted the manuscript. T. Kim provided the data and contributed to the discussion of data. R. Baek, C. Suh, and C. Chung reviewed the manuscript. H. Hee supervised the study. All authors read and approved the final manuscript.

## Pre-publication history

The pre-publication history for this paper can be accessed here:

http://www.biomedcentral.com/1472-6947/12/119/prepub

## Supplementary Material

Additional file 1 Appendix for the investment costs and benefit of Virtual Desktop Infrastructure (VDI) Implementation. Click here for file
