# Supplementary material for: Economic analysis of cloud-based desktop virtualization implementation at a hospital
Source: BMC Med Inform Decis Mak. 2012 Oct 30;12:119. doi: 10.1186/1472-6947-12-119 (PMC3534494; doi:10.1186/1472-6947-12-119)
Supplement: Additional file 1 — Appendix for the investment costs and benefit of Virtual Desktop Infrastructure (VDI) Implementation. [file 1472-6947-12-119-S1.pdf]

Appendix for the investment costs and benefit of Virtual Desktop Infrastructure (VDI) Implementation

Table A shows the investment costs for Virtual Desktop Infrastructure (VDI) implementation at Seoul National University Bundang Hospital (SNUBH). The initial implementation cost is the actual investment amount that arose when VDI was implemented at SNUBH. The operation and maintenance costs include the software annual license (for the use of virtual desktop access), and the operation and maintenance cost was computed as the 7% of the hardware and software cost.

Table A. VDI Investment Costs. **Unit: US$1,000**

Item		Cost
Initial implementation cost	Hardware cost	347
	Software cost	93
	Initial outsourcing implementation cost	159
	Total implementation cost	599
Operation and maintenance cost (five years)	Ongoing support and maintenance cost	62
	Software annual license (for the use of virtual desktop access)	232
	Total operation and maintenance cost	294
Total costs		893

Table B shows the annual gains of four benefit items resulting from the VDI implementation.

Table B. VDI Benefits. **Unit: US$1,000**

Item	Value
A : Benefits due to reduced PC errors and difficulties (consulting rooms)	208
B : Benefits due to reduced PC errors and difficulties (places other than consulting rooms)	27
C : Benefits due to reduced time for application and OS updates	3
D : Benefits due to reduced account management time	0.5
Total increase in revenue (A)	208
Total cost savings (B+C+D)	31
Total benefits	239
